# High-Salt Enhances the Inflammatory Response by Retina Pigment Epithelium Cells following Lipopolysaccharide Stimulation

**DOI:** 10.1155/2015/197521

**Published:** 2015-12-10

**Authors:** Dike Zhang, Chaokui Wang, Shuang Cao, Zi Ye, Bolin Deng, Aize Kijlstra, Peizeng Yang

**Affiliations:** ^1^The First Affiliated Hospital of Chongqing Medical University, Chongqing Key Laboratory of Ophthalmology, and Chongqing Eye Institute, Chongqing, China; ^2^University Eye Clinic Maastricht, Maastricht, Netherlands

## Abstract

High-salt has been shown to play a role in the pathogenesis of autoimmune disease. In this study, we investigated the effect of high-salt on the production of inflammatory mediators by ARPE-19 cells and the possible mechanisms involved. ARPE-19 cells were cultured with LPS in DMEM to which extra NaCl had been added (20 mM and 40 mM). NaCl had no influence on the apoptosis and proliferation of ARPE-19. Addition of 40 mM NaCl significantly induced IL-6 and MCP-1 production but had no effect on IL-8 secretion. High mannitol, as an osmotic stress control, did not affect the secretion of inflammatory mediators by ARPE-19 cells indicating that the effect was not mediated by osmolarity. Coculture of ARPE-19 cells with NaCl resulted in significant increases in the phosphorylation of p38 MAPK, Akt, and NF-*κ*B and an upregulation of the transcription factors NFAT5 and SGK1. High-salt significantly promotes IL-6 and MCP-1 production by ARPE-19 cells and is associated with activation of the p38 MAPK, Akt, and NF-*κ*B pathway and NFAT-SGK1 pathways.

## 1. Introduction

The retinal pigment epithelium (RPE), situated on the outer side of the retina, is a monolayer of cells connected by tight junctions with important functions for the visual system [1]. ARPE-19, a spontaneously arising cell line of RPE, has been extensively used in the past decades to investigate the role of this cell layer in the pathogenesis of a number of diseases including age-related macular degeneration (AMD), vitreoretinopathy, and uveitis [2–5]. The major inflammatory cytokines produced by ARPE-19 in response to various stimuli are interleukin-6 (IL-6), interleukin-8 (CXCL8, IL-8), and monocyte chemoattractant protein-1 (CCL2, MCP-1) [6, 7]. IL-6 is a proinflammatory cytokine that plays an important role in intensifying the intraocular immune and inflammatory response [8–11]. IL-8 and MCP-1 are important chemoattractants of neutrophils, lymphocytes, and monocytes, causing these cells to infiltrate into intraocular tissues during inflammatory retinal disease [12–14].

Both genetic [15] and environmental [16, 17] factors are considered to play a role in the pathogenesis of intraocular inflammation. The role of environmental factors has mainly focused on a role of infections in the development of autoimmune or autoinflammatory uveitis and as yet little attention has been paid to dietary factors [18]. Evidence is now emerging that a high-salt diet may be involved in the pathogenesis of autoimmune disease [19]. Recent studies have shown that high-salt could aggravate the severity of experimental autoimmune encephalomyelitis (EAE) by inducing a Th17 cell immune response, whereby the effect of high-salt on Th17 cell polarization was mediated by activating the p38/MAPK pathway via nuclear factor of activated T cells 5 (NFAT5) and serum/glucocorticoid-regulated kinase 1 (SGK1) [19, 20]. Case-control studies showed that high-salt intake in smokers was associated with the risk of rheumatoid arthritis and a higher salt intake is also associated with increased clinical and radiological disease activity in patients with multiple sclerosis [21]. Whether high-salt affects autoimmune diseases of the eye such as uveitis is not yet known. Since RPE cells play an important role as cellular mediators of the intraocular inflammatory response we decided to investigate the effect of high-salt on the cytokine release by these cells. Our results revealed that high-salt significantly promoted the secretion of IL-6 and MCP-1 by ARPE-19 cells. The high-salt induced release of IL-6 and MCP-1 by ARPE-19 cells was associated with the phosphorylation of p38 MAPK, Akt, and NF-*κ*B and an upregulation of the transcription factors NFAT5 and SGK1.

## 2. Material and Methods

### 2.1. Reagents

Sodium chloride (Chuandong Chemical Group Co., Ltd., Chongqing, China) with a purity of 99.5% was dissolved in deionized water as a stock solution at a concentration of 4000 mM (234 mg/mL). Either 5 *μ*L or 10 *μ*L of this stock solution was added per mL to the cell cultures described below (20 mM or 40 mM additional NaCl). LPS was obtained from Sigma-Aldrich (St. Louis, MO). The ELISA kits to measure IL-8, IL-6, and MCP-1 were purchased from R&D Systems. The annexin V-FITC apoptosis/necrosis detection kit was from KeyGEN Biotech (Nanjing, China). RNA was isolated from ARPE-19 cells using an RNeasy Mini Kit which was purchased from QIAGEN (Valencia, CA); the reverse transcription system and SYBR Green master mix for real-time PCR were from TAKARA (Dalian, China). Antibodies against p38, NF-*κ*B, Akt, JNK, and ERK1/2 were obtained from BD Biosciences (Sunnyvale, CA). A cell counting kit (CCK-8) was from Dojindo (Kumamoto, Japan). Mannitol was obtained from Kelun Pharmaceutical Co., Ltd. (Sichuan, China).

### 2.2. Cell Culture

ARPE-19 was obtained from the American Type Culture Collection (ATCC). Cells were cultured in medium (Dulbecco's modified Eagle's medium: nutrient mixture F12 (DMEM/F12), 1 : 1; Invitrogen, Carlsbad, CA) with 10% fetal bovine serum (FBS, Invitrogen), 100 U/mL penicillin, and 100 ng/mL streptomycin. This medium contains an average of 120 mM NaCl according to the manufacturer. The cells were incubated in a humidified 5% CO_2_ atmosphere at 37°C and passaged every 5 to 7 days. After reaching confluence, the cells were detached with trypsin-EDTA solution, diluted 1 : 3 to 1 : 4, and plated into Corning flask (Corning, Lowell, MA) for subculture at 1.2 × 10^6^ cells/flask and cultured in DMEM/F12 with 10% FBS. The ARPE-19 cells used in the experiments were confluent.

### 2.3. Cell Apoptosis Array

Before stimulation, ARPE-19 cells were serum-starved for 24 hours in serum-free DMEM/F12. Cells were stimulated with 100 ng/mL LPS in the presence or absence of 20 mM or 40 mM extra NaCl for 24 h and then were harvested, centrifuged, washed with PBS, and resuspended with Binding Buffer (KGA108, KeyGEN Biotech, Nanjing, China). Annexin V and PI were added and incubated at room temperature for 15 minutes; cells were analyzed using FACS Aria (BD Biosciences).

### 2.4. Cell Proliferation Assay

The effect of NaCl on ARPE-19 proliferation was detected by CCK-8 according to the manufacturer's instructions. The cells were seeded on 96-well cell culture plate at a density of 5 × 10^3^ cells/well and incubated for 4 days and then incubated in serum-free DMEM and stimulated with or without an extra addition of NaCl (20 mM, 40 mM) in the presence of LPS for 24 h, 48 h, and 72 h, respectively. CCK-8 solution was added to each well of ARPE-19 at each time point, and the cells were further incubated for 2 h at 37°C. The optical density was read at 450 nm using a microplate reader (Molecular Devices, Sunnyvale, CA).

### 2.5. Cytokine ELISA

The levels of IL-6, IL-8, and MCP-1 in supernatants were detected using human ELISA development kits (R&D Systems, Minneapolis, MN) according to the manufacturer's instructions.

### 2.6. Flow Cytometry Analysis

Confluent ARPE-19 cells were cultured with serum-free DMEM/F12 at 37°C in 5% CO_2_ for 24 h. Then the cells were incubated in medium containing additional 20 mM NaCl or 40 mM NaCl in the presence of 100 ng/mL LPS for 20 min. Cells were harvested and stained for intracellular phosphorylated ERK-1/2, P38, Akt, JNK, and NF-*κ*B using BD Phosflow antibodies according to the protocol of the manufacturer (BD Biosciences). Before detection, ARPE-19 cells were fixed in BD Phosflow Fixation Buffer (BD Biosciences) for 10 min at 37°C and permeabilized in BD Phosflow Perm Buffer (BD Biosciences) for 30 min on ice. Anti-phospho-Akt-Alexa Fluor 488, anti-phospho-NF-*κ*B-Alexa Fluor 488, anti-phospho-p38-PE, anti-phospho-ERK-PE, and anti-phospho-JNK-PE (BD Biosciences) were used to stain the cells. Isotype-matched irrelevant Abs were used as controls. Phosphorylation of the five proteins for the various ARPE-19 cell treatment groups was evaluated by flow cytometry and expressed as mean fluorescence intensity (MFI).

### 2.7. Real-Time PCR

Confluent ARPE-19 cells were cultured with serum-free DMEM for 24 h, and then the cells were stimulated with or without NaCl in the presence of LPS for 3 h. RNA was isolated using TRIzol reagent and reverse transcribed to cDNA using Strand cDNA Synthesis Kit (TAKARA, Dalian, China). The primers (NFAT5: forward, 5′-GCAATGGTGATGGAGATGC-3′; reverse, 5′-CTGCTGGTAAACTGGCGATT-3′; SGK-1: forward, 5′-AACACAACAGCACAACATCCA-3′; reverse, 5′-CACCACCAGTCCACAGTCCT-3′; beta-actin: forward, 5′-GGATGCAGAAGGAGATCACTG-3′; reverse, 5′-CGATCCACACGGAGTACTT-3′) were purchased from Sangon Biotech (Shanghai, China). mRNA expression was determined by real-time PCR using the SYBR Green master mix under standard thermocycler condition. Data were collected and quantitatively analyzed on a sequence detection system (ABI Prism 7500, Applied Biosystems). Gene expression was normalized relative to the expression of *β*-actin using methods described elsewhere [22].

### 2.8. Statistical Analysis

Paired-samples* t*-test and Wilcox matched-pairs test were applied using SPSS 19. Data were expressed as mean ± SD. *P* < 0.05 was considered significant for all experiments.

## 3. Results

### 3.1. NaCl Had No Influence on Apoptosis and Proliferation of ARPE-19 Cells following Stimulation with LPS

**Figure 1:**
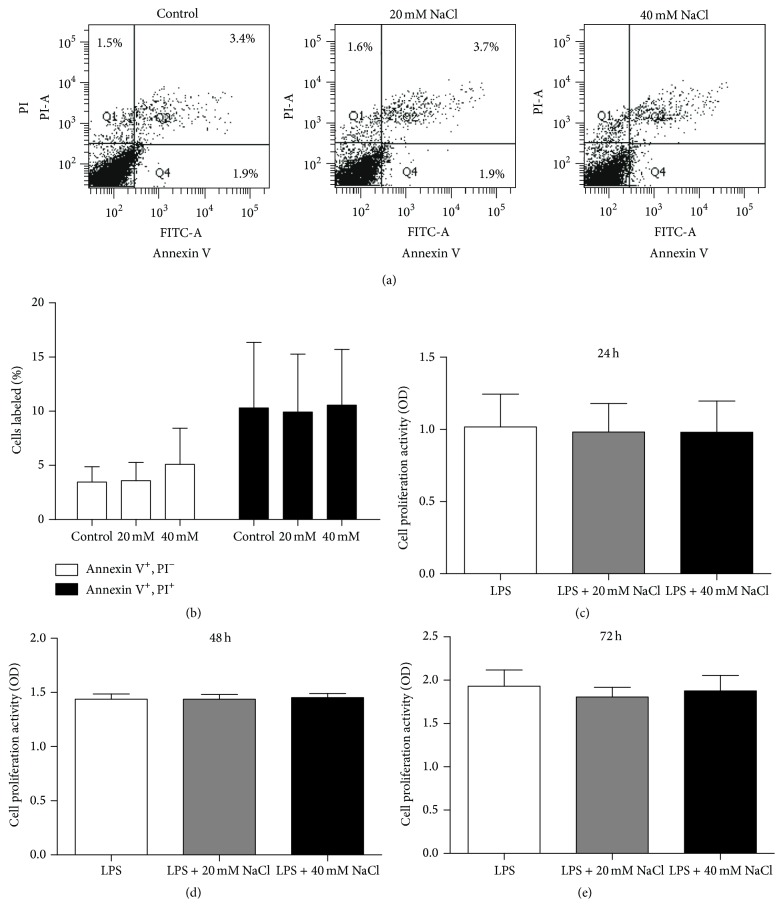
NaCl had no influence on the apoptosis and proliferation of ARPE-19 cells. ARPE-19 cells were stimulated with LPS in the absence or presence of extra additions of NaCl (20 mM, 40 mM) to the culture medium for 24 h. Cells were stained with annexin V and PI for FACS analysis. (a) Apoptotic cells (annexin V^+^ PI^−^) are shown in the Q4 area; late apoptotic cells (annexin V^+^ PI^+^) are shown in the Q2 area; necrotic cells (annexin V^−^ PI^+^) are shown in the Q1 area. (b) The percentages of cells labeled as annexin V^(+)^ PI^(−)^ and annexin V^(+)^ PI^(+)^ were employed for analysis. The data are expressed as means ± SD of three independent experiments and there were no significant differences between the groups, *n* = 7. To detect the proliferation of ARPE-19, the cells were plated with medium alone or medium with extra NaCl (20 mM or 40 mM) for 24 h (c), 48 h (d), and 72 h (e) following LPS stimulation and then measured using the CCK-8 method. Data shown are the mean ± SD of the ratio for light absorbance at 450 nm. Results are representative of three separate experiments, *n* = 6. Paired-samples* t*-test (when the difference between the two tested groups conforms to normal distribution) or Wilcoxon matched-pairs test (when the difference between the two tested groups does not conform to normal distribution) was used for statistical analyses for LPS control versus LPS + 20 mM NaCl or LPS + 40 mM NaCl in each group.

To investigate the effect of NaCl on the apoptosis of ARPE-19 cells, we cultured ARPE-19 cells with an extra addition of NaCl to the culture medium (20 mM and 40 mM), which mimicked high-salt conditions in the interstitium of animals [23], or with placebo in the presence of LPS for 24 h, and then harvested the cells for FACS analysis. The results showed that additional NaCl had no influence on the apoptosis of ARPE-19 cells at a salt concentration of 20 mM or 40 mM (Figures 1(a) and 1(b)).

To exclude the possibility that the effect of the high-salt on the production of inflammatory cytokines by ARPE-19 cells was caused by cytotoxicity of NaCl, we performed CCK-8 assays in preconfluent ARPE-19 cells treated with NaCl (20 mM and 40 mM) for 24 h, 48 h, and 72 h (Figures 1(c), 1(d), and 1(e)). The results showed that the extra addition of NaCl at the concentration of 20 mM and 40 mM did not affect the proliferation of ARPE-19 cells at any given point in time. Therefore, in the subsequent experiments, we used the extra additions of 20 mM and 40 mM of NaCl to investigate the effect of salt on cytokine secretion by ARPE-19 cells and the mechanisms involved.

### 3.2. NaCl Promoted the Secretion of IL-6 and MCP-1 but Not IL-8 by ARPE-19 Cells

**Figure 2:**
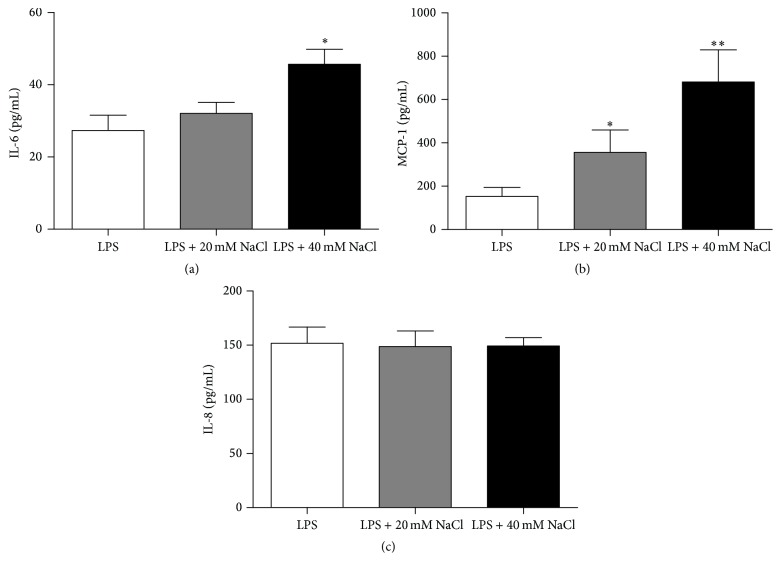
NaCl promotes the secretion of IL-6 and MCP-1 by ARPE-19 cells but had no effect on the production of IL-8. Cells precultured with serum-free medium for 24 h and then stimulated by LPS and cultured with medium alone or medium with an extra addition of NaCl (20 mM and 40 mM) for 24 h. The concentration of IL-6 ((a) *n* = 7), MCP-1 ((b) *n* = 8), and IL-8 ((c) *n* = 8) in cell culture supernatants was measured by ELISA. ^*∗*^ *P* < 0.05 and ^*∗∗*^ *P* < 0.01 for comparison with control and NaCl-treated ARPE-19 cells. The data are expressed as mean ± SD of three independent experiments. Paired-samples* t*-test (when the difference between the two tested groups conforms to normal distribution) or Wilcoxon matched-pairs test (when the difference between the two tested groups does not conform to normal distribution) was used for statistical analyses for LPS control versus LPS + 20 mM NaCl or LPS + 40 mM NaCl in each group.

It has been shown that ARPE-19 cells readily secrete IL-6, IL-8, and MCP-1 following stimulation with LPS. We first investigated whether high-salt had an influence on the secretion of these cytokines by ARPE-19 cells. The results showed that NaCl could significantly induce IL-6 secretion at 40 mM (Figure 2(a)) and that it could also significantly promote the production of MCP-1 by ARPE-19 cells at both 20 mM and 40 mM (Figure 2(b)). However, NaCl had no influence on IL-8 secretion at both concentrations used (Figure 2(c)).

**Figure 3:**
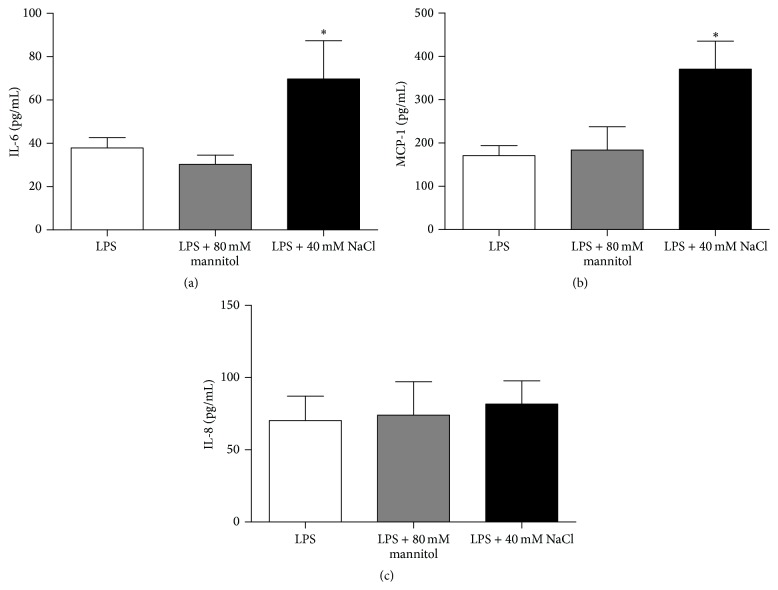
Osmotic stress by itself had no effect on the secretion of cytokines by ARPE-19 cells. Cells were precultured with serum-free medium for 24 h and then stimulated by LPS and cultured with medium alone, medium containing 80 mM mannitol, or medium with extra 40 mM NaCl for 24 h. The secretion of IL-6 (a), MCP-1 (b), and IL-8 (c) was measured by ELISA. The data are expressed as means ± SD of three independent experiments. ^*∗*^ *P* < 0.05 compared to the value of control, *n* = 8. Paired-samples* t*-test (when the difference between the two tested groups conforms to normal distribution) or Wilcoxon matched-pairs test (when the difference between the two tested groups does not conform to normal distribution) was used for statistical analyses for LPS control versus LPS + 80 mM mannitol or LPS + 40 mM NaCl in each group.

We then examined whether the effect of high-salt on cytokine secretion by ARPE-19 cells was due to the osmolarity of high-salt. As the osmolarity of 80 mM of mannitol equals the osmolarity of 40 mM of NaCl, we examined the effect of the addition of 80 mM mannitol on the cytokine secretion by ARPE-19 cells. We found that mannitol had no effect on the production of IL-6, MCP-1, and IL-8 at 80 mM (Figures 3(a), 3(b), and 3(c)). Together, these results suggest that the high-salt could induce the inflammatory response in ARPE-19 cells and that osmotic stress alone did not mediate this process.

### 3.3. The Proinflammatory Effect of NaCl on ARPE-19 Was Associated with the Upregulation of Phosphorylated p38, Akt, and NF-*κ*B and the Increased Expression of NFAT5 and SGK1

**Figure 4:**
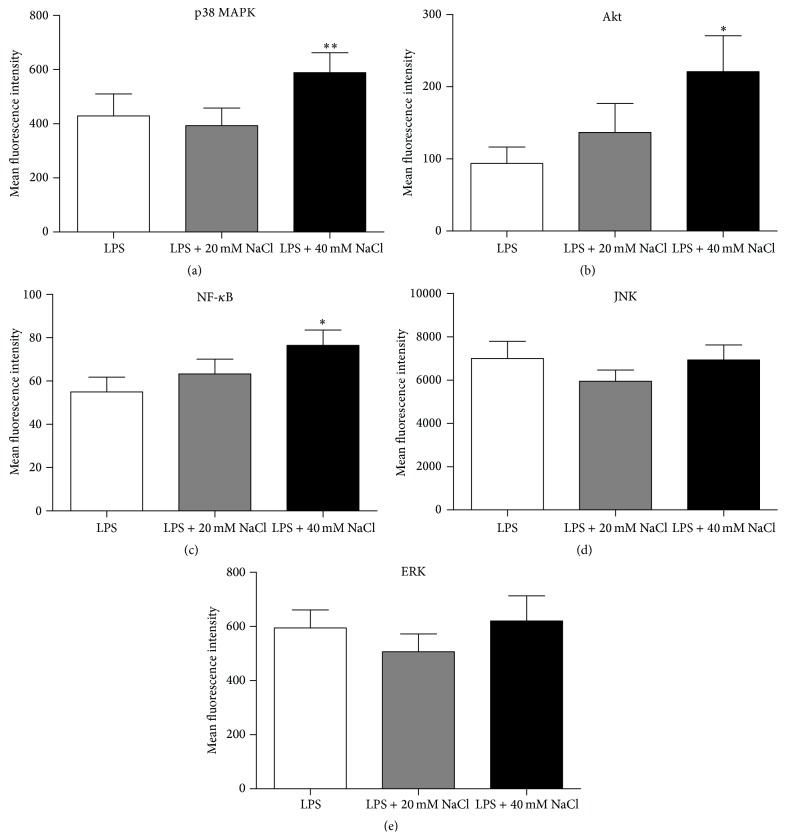
NaCl enhanced the phosphorylation of p38 MAPK, Akt, and NF-*κ*B but had no influence on the phosphorylation of JNK and ERK1/2 in ARPE-19 cells. ARPE-19 cells were cultured with LPS and different additions (20 mM or 40 mM) of NaCl for 20 min. The levels of intracellular phosphorylated signaling molecules in permeabilized ARPE-19 cells were measured by flow cytometry. The data showed the MFI of p38 MAPK ((a) *n* = 7), Akt ((b) *n* = 8), NF-*κ*B ((c) *n* = 13), JNK ((d) *n* = 10), and ERK1/2 ((e) *n* = 7). The results are expressed as mean fluorescence intensity (MFI) ± SD of three independent experiments. ^*∗*^ *P* < 0.05 and ^*∗∗*^ *P* < 0.01 compared to the control. All results are analyzed following three separate experiments. Paired-samples* t*-test (when the difference between the two tested groups conforms to normal distribution) or Wilcoxon matched-pairs test (when the difference between the two tested groups does not conform to normal distribution) was used for statistical analyses for LPS control versus LPS + 20 mM NaCl or LPS + 40 mM NaCl in each group.

To examine the mechanism whereby NaCl induces the production of IL-6 and MCP-1, confluent ARPE-19 cells stimulated with LPS were incubated with or without high-salt (20 mM or 40 mM) for 20 min. The cells were then harvested to measure the level of phosphorylated ERK-1/2, P38, Akt, JNK, and NF-*κ*B by FACS. The results showed that NaCl significantly induced the phosphorylation of P38, Akt, and NF-*κ*B at 40 mM (Figures 4(a), 4(b), and 4(c)). It had no effect on the level of phosphorylated JNK and ERK-1/2 as compared to controls (Figures 4(d) and 4(e)).

**Figure 5:**
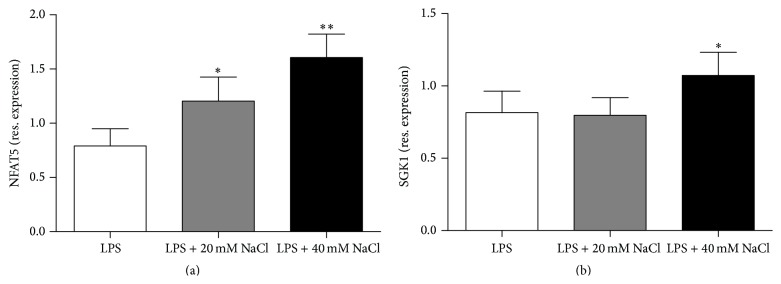
NaCl promoted the expression of NFAT5 and SGK1 on mRNA level in ARPE-19 cells. Cells were stimulated by LPS and incubated with medium alone or additional different concentrations (20 mM or 40 mM) of NaCl for 3 h following preculturing in serum-free medium. Then RNA was isolated and the expression of NFAT5 (a) and SGK1 (b) was measured by RT-PCR. ^*∗*^ *P* < 0.05 and ^*∗∗*^ *P* < 0.01 compared to the control. All results are analyzed following three separate experiments, *n* = 7. Paired-samples* t*-test (when the difference between the two tested groups conforms to normal distribution) or Wilcoxon matched-pairs test (when the difference between the two tested groups does not conform to normal distribution) was used for statistical analyses for LPS control versus LPS + 20 mM NaCl or LPS + 40 mM NaCl in each group.

It has been reported that the inducible effect of high-salt on Th17 cells was mediated by activating the P38 MAPK-NFAT5-SGK1 pathway [19, 20, 24]. In the following experiment we examined the effect of the addition of 40 mM NaCl on the mRNA expression of the downstream translator, NFAT5 and SGK1. The results showed that high-salt increased the gene expression of NFAT5 and SGK1 in ARPE-19 cells (Figures 5(a) and 5(b)).

## 4. Discussion

In this study we show that high-salt significantly stimulates the release of IL-6 and MCP-1 by human ARPE-19 cells. The high-salt induction of IL-6 and MCP-1 was associated with the phosphorylation of p38 MAPK, Akt, and NF-*κ*B and an upregulation of the transcription factors NFAT5 and SGK1. The concentration of additional NaCl we used in our studies was hyperosmolar (20 mM and 40 mM) and resembles interstitial fluid values found in animals fed a high-salt diet [23]. The addition of 20 mM and 40 mM NaCl was tolerated by ARPE-19 cells and had no impact on proliferation or apoptosis of the cells. Although the role of osmotic stress in RPE cell function has been widely studied in the past [25], its effect on the release of inflammatory cytokines has not yet been addressed. Our data are in agreement with earlier studies showing that high-salt can induce IL-6 and MCP-1 by monocytes [26, 27].

High-salt intake is recognized as an important global health issue, especially in view of the fact that many commercially available food items often contain more than 100 times higher salt in comparison to homemade food [28–30]. Epidemiological studies suggest that high-salt intake is associated with an increased risk of multiple sclerosis [31], chronic kidney disease [32], diabetes [33], and chronic heart failure [34]. Long term clinical intervention studies have not yet been reported but experimental animal models have shown that high-salt can aggravate the severity of EAE by inducing Th17 cells [19, 20, 24]. The effect of high-salt on experimental uveitis models or on clinical uveitis has not yet been reported. Breakdown of the blood retinal barrier is known to induce osmotic stress in the posterior segment of the eye, which can affect RPE cell function [25]. This osmotic stress is mainly due to protein extravasation and differs from our model where we investigated salt hypertonicity. Osmotic stress by itself did not lead to a stimulation of the RPE cells since an equimolar concentration of mannitol did not have an effect. This indicates that osmotic stress in combination with the local presence of a high level of sodium ions is responsible for the effects observed. Whether lower doses of NaCl would lead to a stimulated RPE response in situations with a plasma protein related osmotic stress deserves further study.

RPE cells are known to produce only a small amount of cytokines without any stimulation but rapidly respond to certain inflammatory stimuli such as LPS or IL-1 to produce a variety of cytokines, including IL-6, MCP-1, and IL-8 [35–38]. In the studies described here, we chose to use LPS as a stimulus and compared the responses of the RPE cells in the absence or presence of varying amounts of NaCl.

IL-6 has a variety of proinflammatory biological activities and contributes to the pathogenesis of many ophthalmologic disorders including age-related macular degeneration [39], diabetic retinopathy [40], and uveitis [41]. MCP-1 has been shown to be the major chemokine for monocyte infiltration during inflammatory retinal disease [42–44]. Our results suggest that high-salt could contribute to posterior segment inflammation of the eye by enhancing the production of inflammatory cytokines (IL-6 and MCP-1) by RPE cells. We did not find a detectable effect of high-salt on the production of IL-8, whereas a previous study showed that a similar concentration of 40 mM of high-salt as used in our study was able to induce IL-8 production by peripheral blood mononuclear cells (PBMCs) [45]. This discrepancy may be due to the different type of cells used in the experiments. We used an ELISA to study cytokine production, but to strengthen our findings, RT-PCR and flow cytometry might be used in further studies.

It has been shown that LPS-induced signaling cascades involve members of the Ser/Thr protein kinase family including P38 MAPK, ERK1/2, and JNK, which mediate the effect of various extracellular stimuli on biological processes such as cytokine secretion, proliferation, differentiation, and cell death [46–50]. We found that high-salt induced phosphorylation of p38 MAPK in ARPE-19 cells following a combined stimulation with LPS, whereas it did not influence the phosphorylation of JNK and ERK1/2.

Akt is a family of serine/threonine kinases that contains a pleckstrin homology (PH) domain. It has been reported that the Akt pathway could regulate the production of inflammatory mediators in response to inflammatory stimuli and that it plays an important role in cell survival [51–53]. We found that high-salt can activate the phosphorylation of Akt in ARPE-19 cells, suggesting that the Akt pathway may also be involved in the high-salt induction of IL-6 and MCP-1 by these cells.

NF-*κ*B is a ubiquitously expressed transcription factor that regulates the expression of approximately 200–300 genes. The activation of NF-*κ*B plays an important role in the induction of inflammatory cytokines during the inflammatory response [54, 55]. Our results showed that high-salt enhanced the phosphorylation of NF-*κ*B in LPS-treated ARPE-19 cells, suggesting that NF-*κ*B activation is involved in the high-salt response and that it may be responsible for the expression of inflammatory mediators (IL-6 and MCP-1).

Further blocking experiments using antagonistic agents and gene knockout experiments that interfere with the signaling pathways should be performed to verify the effect of p38 MAPK, Akt, and NF-*κ*B. We used FACS analysis to investigate the phosphorylation events in these pathways, but Western blotting could also be used to confirm these findings and to verify the effect of IbK and other phosphorylation sites (e.g., s475 or Thr378 for Akt and others) in the signaling pathways.

Recent studies have reported that high-salt could induce Th17 cell polarization by activating the p38/MAPK pathway involving the downstream transcription factors NFAT5 and SGK1 in mouse CD4^+^T cells [19, 20]. Our study showed that high-salt also induced the gene expression of NFAT5 and SGK1 in ARPE-19 cells, which is consistent with the results observed in CD4^+^T cells in mouse studies. These data suggest that p38 MAPK-NFAT5-SGK1 activation is also involved in the induction of inflammatory cytokines by ARPE-19 cells in response to high-salt.

In conclusion, our studies showed an aggravating effect of high-salt on the inflammatory response by ARPE-19 cells. Additionally, we found that the induction of IL-6 and MCP-1 by ARPE-19 cells was associated with the phosphorylation of p38 MAPK, Akt, and NF-*κ*B and the upregulation of transcription factors NFAT5 and SGK1. Our data indicate that, at least partly, high-salt intake may increase the risk of developing inflammatory diseases in the posterior segment of the eye. Further confirmatory studies are needed to investigate the effect of high-salt on cytokine production by primary human RPE cells and whether patients suffering from autoimmune or autoinflammatory eye diseases should limit their salt intake.

## 5. Conclusion

These results suggest that local high-salt may contribute to the intraocular inflammatory response by promoting proinflammatory cytokine production.
